# Prebiotic Mannan Oligosaccharide Pretreatment Improves Mice Survival Against Lethal Effects of Gamma Radiation by Protecting GI Tract and Hematopoietic Systems

**DOI:** 10.3389/fonc.2021.677781

**Published:** 2021-06-24

**Authors:** Sweta Sanguri, Damodar Gupta

**Affiliations:** Division of Metabolic Cell Signaling Research, Institute of Nuclear Medicine & Allied Sciences, Delhi, India

**Keywords:** total body irradiation, gastrointestinal syndrome, hematopoietic syndrome, radiation countermeasure agents, mannan oligosaccharide, prebiotics, mitochondria

## Abstract

Total body irradiation (TBI) results in critical injuries in a dose dependent manner that primarily damages highly proliferating tissues including hematopoietic stem cells (HSCs) and intestinal crypt stem cells *etc.* This may result in hematopoietic syndrome leading to bone marrow failure and gastrointestinal syndrome leading to chronic intestinal functional alterations. Death results from the gastrointestinal syndrome due to sepsis, bleeding, dehydration, and multi-system organ failure. We demonstrate that the prebiotic mannan oligosaccharide (MOS) pretreatment substantially prolongs survival in both male and female mice when administered 2 h prior to radiation either through oral or intraperitoneal route. The radioprotective efficacy of MOS was found to be age dependent and improves survival even in aged mice (12–13 months old). MOS pretreatment effectively abrogates radiation-induced hematopoietic injury and accelerates recovery of lymphocytes and WBCs and alleviates depletion of circulatory blood cells. Results also illustrate that MOS pretreatment abolishes crypt cell death and denudation of villi in comparison to the respective irradiated animals and ameliorates the overall radiation-induced damage to the GI system. MOS pretreatment facilitates intestinal recovery leading to enhanced animal survival demonstrating its protection efficacy against TBI induced mortality. Moreover, MOS pretreated animals show signs of accelerated recovery in terms of severity of radiation sickness symptoms including weight loss and completely abolish TBI associated mortality.

## Introduction

Development of safe and effective radiation countermeasure approaches, including radio-protectors, mitigators, and therapeutics, are crucial and vital for the management of prospective disaster scenarios comprising low and high dose radiation exposure (1). Radiation exposure can result in mortality or can cause long-term adverse health effects depending upon the exposure dose, dose rate, and time of exposure (2). Acute radiation syndrome (ARS) arises mainly due to the intense enormity of injury to the actively proliferating cells including hematopoietic stem cells (HSCs) and intestinal crypts stem cells *etc.* resulting in hematopoietic acute radiation syndrome (H-ARS; leading to bone marrow failure) and gastrointestinal acute radiation syndrome (GI-ARS; leading to chronic intestinal functional alterations) respectively. High dose TBI can result in neurovascular acute radiation syndrome (NV-ARS) leading to death generally within 24–48 h. NV-ARS is considered fatal due to irreversible damage to organs. Therefore, only H-ARS and GI-ARS are being focused for the development of radiation countermeasures (3). Radiation exposure induces damage to the intestinal crypt cells in the gastrointestinal tract, compelling crypt cells to undergo apoptosis within hours after ionizing radiation (IR) exposure (4). Since GI tract is an extremely rapid cell turnover tissue, depletion of crypt cells results in homeostasis imbalance, and restoration of intestinal villi is impaired, leading to loss of villi cellularity and denudation of the villi (5, 6). Consequently, other morphological changes including disruption of the epithelial barrier manifest along with compromised functional capacity leading to chronic intestinal functional alterations comprising GI-ARS. Hence, it is obligatory to identify efficient, medically safe, and affordable radiation countermeasure agents (RCAs) that can potentially prevent or manage both H-ARS and GI-ARS.

Toll-like receptor (TLR) family is one of the imperative components of innate immune system, which is the first line of defense against microbial invasion (7). TLRs can recognize pathogens, molecular patterns and/or danger signals and induce immune signaling for evasion of infection. Stimulation of TLR2, 4, 5 or 9 by corresponding ligand(s) has been shown to reduce radiation-induced cell death in crypt cells, resulting in improved radiation-induced GI-ARS symptoms (7). Consequently, different TLR ligands are currently under different stages of development as RCAs for ARS. Manipulation of TLR functions has been shown to activate NF-*κ*B pathway and reduce radiation-induced cell death. Entolimod (CBLB502, a TLR5 agonist) has been granted both Fast Track and Orphan Drug status by the US FDA during or after a radiation disaster to reduce risk of death. Entolimod has been shown to reduce radiation damage to both hematopoietic (HP) and gastrointestinal (GI) tissues and improve tissue regeneration (2, 8, 9).

Mannan oligosaccharide (MOS) is a TLR agonist and is used as prebiotic nutritional supplement in several living organisms including farm animals, cattle, pigs, dogs, chicken, fishes *etc.* for its gastrointestinal and immunological responses (10, 11). MOS is reported to improve health, growth status, and overall performance in animals (12–14). It is known to support the gut microflora and stimulates epithelial barrier structure and functionality of intestinal mucosa (12). MOS is shown to increase microvilli surface area and goblet cell numbers in small intestine of animals (15). It stimulates the immune system of the host and has adsorbent capacity against toxins and it is non-toxic when administered orally, even in very large concentration (16, 17). Under *in vitro* conditions, we have demonstrated that MOS mediates alteration in mitochondrial physiology in immortalized normal cells and offer advantages in reducing biological effects of *γ*-radiation *in vitro* and thereby enhances cell survival (18, 19). TLRs have been reported to express in humans and mice (3, 18, 20), we intend to utilize the benefits of MOS supplementation against radiation induced ARS. Since mitochondrial respiratory activity is reported to decline during the natural aging process, we investigated the effects of MOS pretreatment (50 mg/kg/B.W i.p. and 200 mg/kg B.W. orally; 2 h prior to irradiation) on IR-induced injury in different age groups of BALB/c mice at lethal (7.5 Gy) and sub-lethal (3 and 5 Gy) doses of TBI. In our preliminary experiments, we administered MOS intraperitoneally (50 mg/kg/B.W i.p; 2 h prior to irradiation) and observed 100% survival advantage in mice at lethal dose of TBI. MOS as a prebiotic is non-toxic orally and has beneficial effects in overall health of an organism. Remarkably, MOS oral pretreatment (200 mg/kg B.W. orally; 2 h prior to TBI; 7.5 Gy) also confers 100% radiation protection at lethal dose of TBI despite having a different biodistribution pattern than through intraperitoneal route as studied earlier (14). TBI instigates bone marrow (BM) suppression resulting in loss of circulating blood cells. It also causes significant loss of viable crypt cells in the intestine, perturbs villus structure, disrupts mucosal layer integrity thereby compromising proper absorption of essential nutrients. The immune-suppression and thrombocytopenia associated with the H-ARS favor opportunistic infections and hemorrhage. MOS pretreatment to mice effectively minimizes radiation-induced hematopoietic and gastrointestinal injury, accelerates recovery of circulating blood cells, minimizes oxidative damage to important cellular biomolecules, restores intestinal integrity and consequently abrogates TBI-induced lethality.

## Materials and Methods

### Chemicals

All chemicals used in the study were of analytical grade and were either procured from an Indian manufacturer (SRL India, HiMedia chemicals) or obtained from Sigma Aldrich (St Louis, MO), Thermo Fisher Scientific Inc (USA) *etc.* Mannan oligosaccharide (MOS), 1,3,5-trihydroxybenzene, phloroglucinol, D-xylose, ethylene diaminetetracetic acid (EDTA), BCA kit, hematoxylin, eosin, thiobarbituric acid, BSA, Mops, Sucrose, TBA, DTNB, NEM, neutral buffered formalin, decalcifying solution-Lite, were obtained from Sigma-Aldrich Chemical Co., St. Louis, MO, USA. Phosphate buffer saline (PBS), acetic acid, hydrochloric acid, and all other chemicals obtained were of analytical grade from SRL India. Reagents for hematology analyzer (Isotonac 3, Hemolynac 5, Hemolyzing Reagent, Hemolynac 3, Cleanac 3) were procured from Nihon Kohden, Japan.

### Mice

Inbred BALB/c mice were obtained from the central experimental animal facility of the Institute of Nuclear Medicine & Allied Sciences (INMAS), Defence Research and Development Organization (DRDO). Both male and female mice of different age groups *viz.* 8–12 weeks, 6–8 months, and 12–13 months old acclimatized and healthy animals were chosen for studies. Mice were housed in the facility maintained at 21 ± 2°C with 50 ± 10% humidity on a 12 h light/dark cycle and were fed standard rodent feed (from Golden Feeds, Delhi, India) and water *ad libitum*. All surviving mice were euthanized at the completion of the observation period.

### Irradiation

Mice were subjected to total body irradiation (TBI) in 60Co Gamma Teletherapy unit (Bhabhatron II, Panacea Medical Technologies, Bangalore, India) at a dose rate of 2.25–2.55 Gy/min. (dose rate of the 60Co Gamma irradiation source was calibrated using physical dosimetry). Mice were subjected to radiation exposure of either 3 or 5 Gy and/or lethal dose of 7.5 Gy separately with or without MOS pretreatment.

### Preparation and Administration of MOS

MOS was dissolved (20 mg/ml stock) in sterile phosphate buffered saline (PBS; 1×) under aseptic conditions. MOS was administered (intra-peritoneally 50 mg/kg body weight or orally 200 mg/kg body weight separately as indicated) 2 h prior to total body irradiation (TBI) in mice for radioprotection efficacy studies.

### Survival Studies

To study radio-protective efficacy of MOS, mice were divided into the following groups: Control (sham irradiated; vehicle treated), MOS alone, Radiation (3 or 5 or 7.5 Gy) alone, and mice that received MOS 2 h before TBI (3 or 5 or 7.5 Gy; n = 8/group). MOS was administered intra-peritoneally (50 mg/kg body weight) or orally (200 mg/kg body weight) 2 h prior to TBI in a single fraction as per groups, as indicated. All animals were weighed, and their well-being was inspected daily from the initiation of treatment to the end of the study.

### Evaluation of Biological Effects of Radiation

To evaluate effects of MOS pretreatment in radiation protection and minimize biological effects of radiation, mice were exposed to different doses of IR (3 and 5 Gy) separately. Mice were divided into the following six groups: Control (sham irradiated, vehicle treated), MOS alone (oral; 20 0mg/kg body weight), radiation alone (3 or 5 Gy alone), mice that received MOS 2 h before 3 or 5 Gy TBI (n = 8/group). MOS was administered orally at 200 mg/kg body weight 2 h prior to TBI (3 or 5 Gy) in a single fraction as per group. The mice were sacrificed on days 3, 7, 15, and 40 as per ethical guidelines (by cervical dislocation), and tissues were collected. The tissues were washed in PBS and either fixed for histology or flashed frozen in liquid N_2_, and stored for further assays.

### Peripheral Blood Analysis

Blood was withdrawn from the retro-orbital plexus and collected in heparinized blood collection tubes (BD Biosciences, San Jose, CA) on days 3, 5, 15, and 21 following various treatments. Blood was mixed gently on a rotary shaker until acquisition and analysis for red blood cells, hemoglobin, platelets, leukocytes, and lymphocytes by using hematology analyzer (MEK-6400; Nihon Kohden, Japan), and data was generated using Data Management Software (DMS-Lite software).

### Histological Examination of Bone Marrow

Mice were euthanized humanely, and femurs were isolated on days 3, 7, 15, and 40-post IR exposure or/and MOS treatment. Histological examination of bone marrow was done as described by Travlos et al. with minor modifications (21). Briefly, femurs were fixed in 10% formalin, neutral buffered (Sigma) for 12–14 h at RT with gentle rocking followed by incubation in decalcifying solution-Lite (Sigma) at RT with gentle rocking for 14–16 h. Femurs were then washed thoroughly in tap water and thereafter processed for dehydration, and paraffin blocks were made. The micro-sections (3 μm) of tissue were prepared and stained with hematoxylin and eosin (H&E). Slides were examined by microscopy, and brightfield image acquisition was done using Cell imager, Optika, Italy.

### Histological Examination of Small Intestine

Histological examination of small intestine was done as described by Morson et al. (22) with minor modifications. Briefly, the mice were euthanized humanely, and intestine of each animal was dissected, washed with PBS to remove intestinal contents (on days 3, 7, 15 and 40-post IR exposure). Following washing, jejunums were fixed in 10% neutral-buffered formalin (Sigma) at 37°C for 24 h. The fixed tissue was thereafter processed for dehydration, and paraffin blocks were made. The micro-sections (5 μm) of tissue were prepared and placed on slides for staining with hematoxylin and eosin (H&E). Slides were examined by microscopy and brightfield image acquisition was done using Cell imager, Optika Italy. Villus height and crypt depth were measured and compared with that of control. Villus height was determined by measuring the distance from the tip of the villus up to the crypt. Spatial scale of the active image was defined and presented in micrometer (calibrated unit, 2.55 pixels/micrometer) by converting pixels to μm using ImageJ software for mac (Version 1.50i, NIH USA).

### Crypt Microcolony Survival Assay

Non-serial transverse sections of jejunum were studied for the number of cells per crypt and surviving crypt per T.S. Each section was separated from the previous one by a minimum of 50 µm of tissue. Surviving crypts with ≥10 cells for each T.S. were counted, and results are expressed as number of surviving crypt/T.S. jejunum (23). A surviving crypt was defined as one that had ten or more tightly and strongly packed H & E stained cells (excluding Paneth cells). Only regions that were orientated correctly and did not contain Payer’s patches were scored (Payer’s patches influence both the number of crypts in a normal circumference and the ability of a crypt to survive insult). Number of cells per crypt was also counted, and results were expressed as number of cells/crypt. Data were pooled from three to four separate T.S. of jejunum from each mouse. Only those crypts which were seen directly against the inner muscle layer were counted. All counts and measurements from each tissue specimen were obtained “blind” from a minimum of four coded sections.

### D-Xylose Absorption Assay

To quantify absorption efficacy of intestine as a physiological indicator of mucosal barrier integrity in mice (n = 8/group) after various treatments, a D-xylose uptake assay was performed at various time points (0, 3, 7, 10, 15 and 40 days post 3, 5, and 7.5 Gy TBI). A 5% w/v solution of D-xylose (100 μl/mouse) in deionized water was administered orally, and blood samples were collected 2 h post administration. Blood was withdrawn from the retro-orbital plexus and collected in microtainer tubes (BD Microtainer Gold tube, BD Biosciences, San Jose, CA). Following 30 min coagulation at room temperature, the sera were well separated from the gel by 10 min-centrifugation at 10,000g, collected and stored at −80°C for later study. Serum D-xylose concentration was determined according to Eberts et al. with minor modifications (24). Then 5 ml phloroglucinol (1,3,5-trihydroxybenzene, Sigma Chemical Co., St. Louis, MO) reagent (0.5 g of phloroglucinol, 100 ml glacial acetic acid, and 10 ml of conc. HCl) was added to 50 μl of plasma. This solution was heated to 100°C in a water bath for 4 min to allow optimum color development. After equilibration to room temperature, sample absorption was measured using spectrophotometer at 554 nm, and D-xylose concentration in each serum sample was calculated using D-xylose standard calibration curve.

### Isolation of Mitochondria

Kidney and liver tissues stored at −80°C were taken out, and homogenate (10%) was prepared in ice-cold isolation medium (0.3 M sucrose, 0.1% BSA, 1 mM EGTA, 5 mM Mops, 5 mM KH_2_PO_4_, pH 7.4) using a Potter Elvjham homogenizer, and mitochondria were isolated using the method of Goel et al. (25). Briefly, the homogenate was centrifuged at 1,000g for 10 min at 4°C. The supernatant was collected and centrifuged at 10,000g for 20 min at 4°C to obtain the mitochondrial pellet. The mitochondrial pellet was washed three times with 50 mM potassium phosphate buffer (pH 7.4) to remove traces of sucrose, and integrity of mitochondria was determined by measuring the monoamine oxidase enzyme activity (26, 27).

### Mitochondrial Lipid Peroxidation

Thiobarbituric acid reactive substances (TBARSs) were measured spectrophotometrically in liver and kidney homogenates as described by (28) with minor modifications. Briefly, mitochondrial protein (4 mg/ml) was mixed with an equal volume of Buege & Aust reagent (TCA, 15% (w/v) in 0.25 M HCl; TBA, 0.37% (w/v) in 0.25 M HCl) and heated for 15 min in boiling water. After cooling, the precipitate was removed by centrifugation at 1,000g in a refrigerated centrifuge (Sigma 3-18K, St. Louis, MO, USA) for 10 min at room temperature. The absorbance of the supernatant was recorded at 532 nm (IMPLEN nanodrop, Germany) against a sample containing reagents but no sample. The concentration of TBARS was determined using an extinction coefficient of 1.56 × 105 mol^−1^ cm^−1^ and results are expressed as nmoles of MDA per mg of mitochondrial protein. Protein concentration in each sample was measured by using BCA kit (Sigma-Aldrich Chemical Co., St. Louis, MO, USA) as per manufacturers’ protocol using bovine serum albumin (BSA) as standard.

### Thiol Estimation

Acid soluble thiol was measured spectrophotometrically in liver and kidney homogenates as described by *Ellman* with minor modifications (29). Briefly, 0.3 ml mitochondrial protein (4 mg/ml) was mixed with an equal volume of SDS (10%) and mixed thoroughly. A 2.4 ml sodium phosphate buffer (5 mM) was added, and the solutions were mixed properly. The background absorbance of the solution was recorded at 412 nm using spectrophotometer. A 0.3 ml DTNB (1 mM) was then added, and the solutions were incubated for 1 h at 37°C, and absorbance was measured at 412 nm. The concentration of sulfhydryls in the sample was determined using molar extinction coefficient of TNB (14,150 M^−1^ cm^−1^), and results are expressed as nmol of TNB per mg of mitochondrial protein. Protein concentration in each sample was measured by using BCA kit (Sigma-Aldrich Chemical Co., St. Louis, MO, USA) as per manufacturers’ protocol using bovine serum albumin (BSA) as standard.

### Statistical Analysis

All the data were analyzed using Graph Pad Prism (version 5.01) and were presented as mean ± standard deviation (SD). Significance of difference between groups was determined by Student’s t-test and one-way or two-way ANOVA with Newman–Keuls’ multiple composite-tests. Survival studies’ data were analyzed using the Kaplan–Meier method followed by Mantel–Cox (log-rank) test for assessment of significant differences. Results were considered significant at P < 0.05.

## Results

### Survival Studies of Mice Following Treatment With MOS

**Figure 1:**
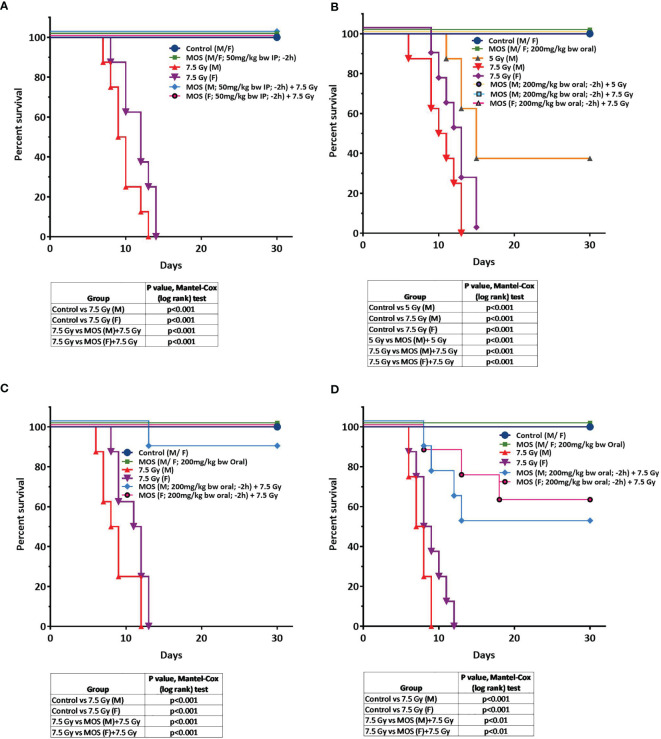
**(A–D)** MOS pretreatment protects mice from TBI-induced lethality. Protective effects of MOS (*i.e.* administered 2 h prior to irradiation) against lethal TBI (7.5 Gy). **(A)** Mice (8 to 12 weeks old, both male and female) were injected MOS (50 mg/kg/B.W.; i.p.) and irradiated 2 h post treatment. **(B)** Protective effects of MOS (*i.e.* administered orally 200 mg/kg B.W.; 2 h prior to irradiation) against lethal TBI (7.5 Gy) and sub-lethal TBI (5 Gy). Mice (8 to 12 weeks old, both male and female) were administered MOS orally and irradiated 2 h post treatment. **(C)** Mice (6**–**8 months old, both male and female) were administered MOS orally (200 mg/kg B.W.) and irradiated 2 h post treatment. **(D)** Mice (12**–**13 months old, male) were administered MOS orally (200 mg/kg B.W. and irradiated 2 h post treatment. Kaplan**–**Meier survival curve depicts the 30-day survival (n = 8 in all the groups). The table summarizes the findings of Mantel**–**Cox (log-rank) test comparing survival responses between un-irradiated and TBI cohorts, TBI and MOS (−2h) +TBI cohorts.

Survival studies performed on Balb C mice showed that all the mice exposed to lethal doses of gamma radiation (7.5 Gy) alone died within 14 days (LD-100/14). Treatment of mice with MOS alone (IP 50 mg/kg BW or oral 200 mg/kg BW) did not influence the survival of mice. Moreover, oral administration of MOS showed no toxicity in terms of survival up to 5 g/kg BW. Pre-irradiation treatment of mice with MOS (−2 h; 50 mg/kg bw; IP) conferred remarkable protection of mice resulting in the 100% survival of mice (both male and female mice, age 8–12 weeks) as observed till 30 days (**Figure 1A**; p < 0.001). Interestingly, pre-irradiation treatment of mice with MOS (−2 h; 200 mg/kg bw; orally) also conferred 100% survival of mice (both males and females, age 8–12 weeks and female, 6–8 months, **Figures 1B, C**; p< 0.001) as observed till 30 days and thereafter also (up to 6 months) with respect to group exposed to radiation (7.5 Gy) alone. The radioprotective efficacy of MOS was found to be age dependent with 90, 60, and 50% survival in 6–8 months male mice, 12–13 months old female mice, and 12–13 months old male mice respectively (**Figures 1C, D**; p < 0.001) observed till 30 days. At 7.5 Gy TBI dose, the radiation alone group experienced absolute mortality and the entire cohort of animals died on (or before) 15–16 days post TBI accompanied with symptoms of radiation sickness such as weight loss, diarrhea, anorexia, and lethargy. The animals treated with MOS prior to TBI showed significantly less severity of symptoms or no symptoms at all. The studies were also performed to measure survival at sub-lethal dose of TBI (5Gy). Exposure of mice to 5 Gy radiation also found to reduce survival by 60% (LD-60/21) with respect to control group. Administration of MOS (male; 200 mg/kg bw orally; −2 h) conferred 100% protection with respect to 5 Gy alone (p < 0.001).

The result suggests that MOS treatment prior to radiation protects animal from deleterious effects of radiation and confers 100% survival when administered from either of the two routes.

### MOS Pretreatment Amends Hematological Indices in the Peripheral Blood Post TBI

**Figure 2:**
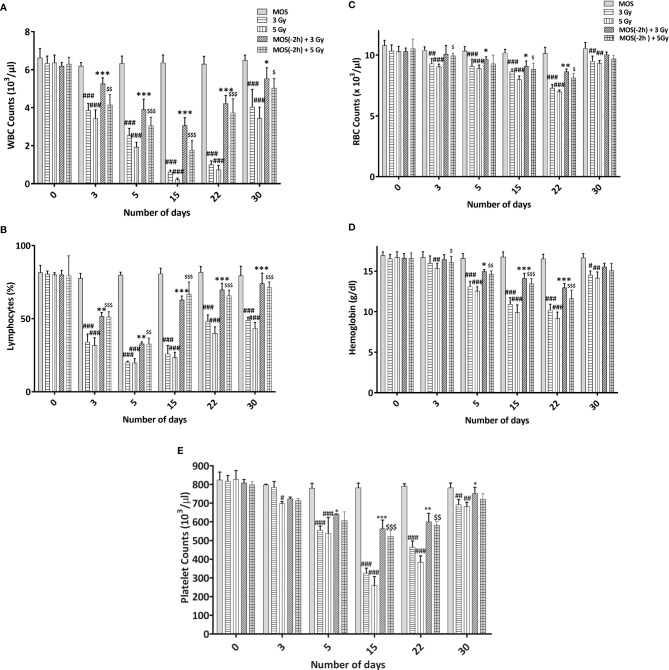
**(A–E)** MOS mediated alterations in the hematological indices in the peripheral blood. Mice were treated with MOS and/or subjected to exposure of radiation (3 and 5 Gy) and assessed for blood cell parameters as described in *Materials and Methods*. Blood counts representing **(A)** WBC, **(B)** lymphocyte, **(C)** RBC, **(D)** hemoglobin, and **(E)** platelets at days 3, 5, 15, 22, and 30 post TBI. (n = 10). Error bars represent mean ± SD, and *p < 0.05, **p < 0.01, and ***p < 0.001, ^#^ *p* < 0.05, ^##^ *p* 0.01, ^###^p < 0.001, ^$^ *p* < 0.05, ^$$^ *p* < 0.01 and ^$$$^ *p* < 0.001 were considered significant, #compared to unirradiated control, *compared to 3 Gy irradiated cohorts and $compared to 5 Gy irradiated cohorts.

The kinetics of hematopoietic recovery in sub-lethally irradiated mice treated with MOS was followed for 30 days post-TBI. MOS significantly increased survival from 60 to 100% in combination (MOS + 5Gy) cohorts compared to the TBI (5 Gy) group. Assessment of the peripheral blood for various hematological parameters exhibited significant decrease in peripheral blood components (**Figures 2A–E**). The results revealed that exposure to TBI significantly decreased the number of circulating leukocytes in animals exposed to TBI (3.87 × 10^3^ ± 0.35 cells/μl, p < 0.001) and 3.43 × 10^3^ ± 0.45, p < 0.001) cells/μl in 3 and 5Gy TBI cohorts respectively) in comparison to 6.63 ×10^3^ ± 0.5 cells/μl in un-irradiated control cohorts, with nadir observed at day 15 post-irradiation (0.62 ×10^3^ ± 0.1 cells/μl, p < 0.001 and 0.217 × 10^3^ ± 0.08 cells/μl, p < 0.001) in 3 and 5Gy TBI, respectively). Decrease in circulating leukocytes can compromise the health status of animals and can result in immunosuppression. Leukocyte levels did not recover till day 30 in the TBI mice. On the contrary, MOS pretreatment augmented the WBC count by accelerated recovery in the leukocyte counts at day 22 (4.23 × 10^3^ ± 0.42 cells/μl, p < 0.001 and 3.74 ×10^3^ ± 0.73 cells/μl, p < 0.001) in MOS +3 Gy and MOS + 5 Gy group respectively), and recovered to almost normal levels by day 30 in animals of both MOS + 3 Gy and MOS + 5 Gy groups. The lymphocyte percentage significantly decreased at day 3 (34.03 ± 5.43%, p < 0.001 and 31.78 ± 5.20%, p < 0.001) and reached their nadir around day 5 (20.20 ± 0.73%, p < 0.001 and 19.82 ± 2.85%, p < 0.001) and did not recover until after day 30 post-TBI [(49.0 ± 1.97%, p < 0.001) and (43.50 ± 3.95%, p < 0.001)], in both 3 and 5 Gy cohorts, respectively (**Figure 2B**). Contrarily, in MOS pretreated cohorts, the initial decrease in lymphocyte percentage was rapid till day 5 post TBI, after which accelerated recovery was observed and approximately normal percentage was reached by day 30 post TBI. Erythrocytes and hemoglobin were slower to reach their nadir (day 22), and both recovered to almost normal levels by day 30 (**Figures 2C, D**) both in TBI and MOS + TBI cohorts. Exposure to TBI in irradiated group resulted in initial gradual decrease in platelet count at day 3 (784.07 × 10^3^ ± 32.42 cells/μl, p = ns and 697 × 10^3^ ± 12.00 cells/μl, p < 0.01) followed by a subsequent sharp decrease at day 5, reached nadir (325.60 × 10^3^ ± 26.66 cells/μl, p < 0.001 and 259.10 × 10^3^ ± 47.92 cells/μl, p < 0.001) at day 15 post TBI, and recovered levels were seen in 3 and 5 Gy irradiated cohorts respectively, by day 30 post TBI (**Figure 2E**). Hematopoietic suppression was significantly lesser in TBI mice pretreated with MOS in all studied parameters. Thus, these results suggest that MOS could be a potential countermeasure for the reduced number of circulating blood cells in irradiated animals.

### MOS Pretreatment Restores Bone Marrow Cellularity in TBI Mice

**Figure 3:**
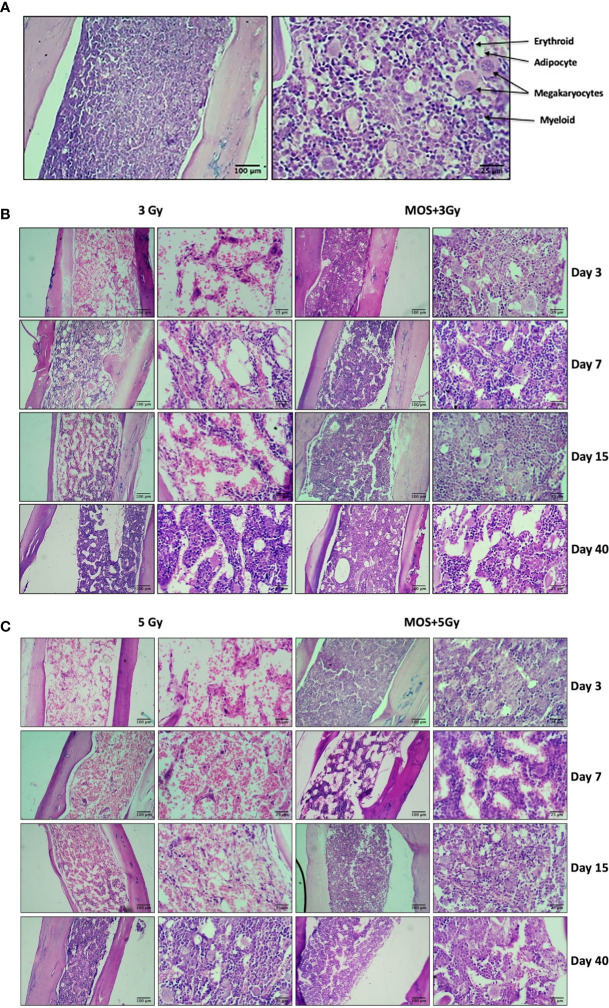
Histological examination of bone marrow. Mice were treated with MOS and/or subjected to TBI (3 and 5 Gy) and assessed for histological examination of small intestine on days 3, 7, 15, and 40-post MOS treatment and/or TBI as described in *Materials and Methods*. **(A)** Representative image of H&E stained femur section of un-irradiated control mice. **(B)** Representative image of H&E stained femur section of 3 Gy TBI and 3 Gy +M mice. **(C)** Representative image of H&E stained femur section of 5 Gy TBI and 5 Gy +M mice.

TBI above a threshold dose induces hematopoietic injury and is characterized by depletion of the bone marrow cellular content (7, 30). Effects of MOS pretreatment on the radiation-induced damage to the bone marrow were analyzed by histological analysis of the mice femurs on days 3, 7, 15, and 40 post TBI (3 and 5 Gy). In TBI mice, hematopoietic stem cells and vasculature are disrupted to a large extent, and results show dramatic decrease in bone marrow cellularity in TBI mice. The bone marrow appears necrotic and highly hemorrhagic, and the marrow space shows high content of RBCs (anuclear pink cells). Scattered islands of hematopoietic cells were found to occupy the marrow cavity with variable cellularity and decreased hematopoiesis, till day 15 post 3 and 5 Gy TBI; (**Figures 3B, C**) in comparison to un-irradiated animals that show hypercellular marrow, with more than 90% cellularity with trilineage hematopoiesis. Erythroid series is visible; myeloid series show complete maturation, and megakaryocytes are adequate in number and morphology; **Figure 3A**. Contrarily, MOS pretreatment abrogated the radiation-induced cellular depletion to a large extent with cell numbers and cellular content almost similar to levels in the control animals, and accelerated recovery. The data corroborates well with peripheral blood analysis and indicate that MOS pretreatment alleviates the TBI-induced BM suppression resulting in enhanced hematopoietic recovery.

### Histological Examination of Small Intestine

**Figure 4:**
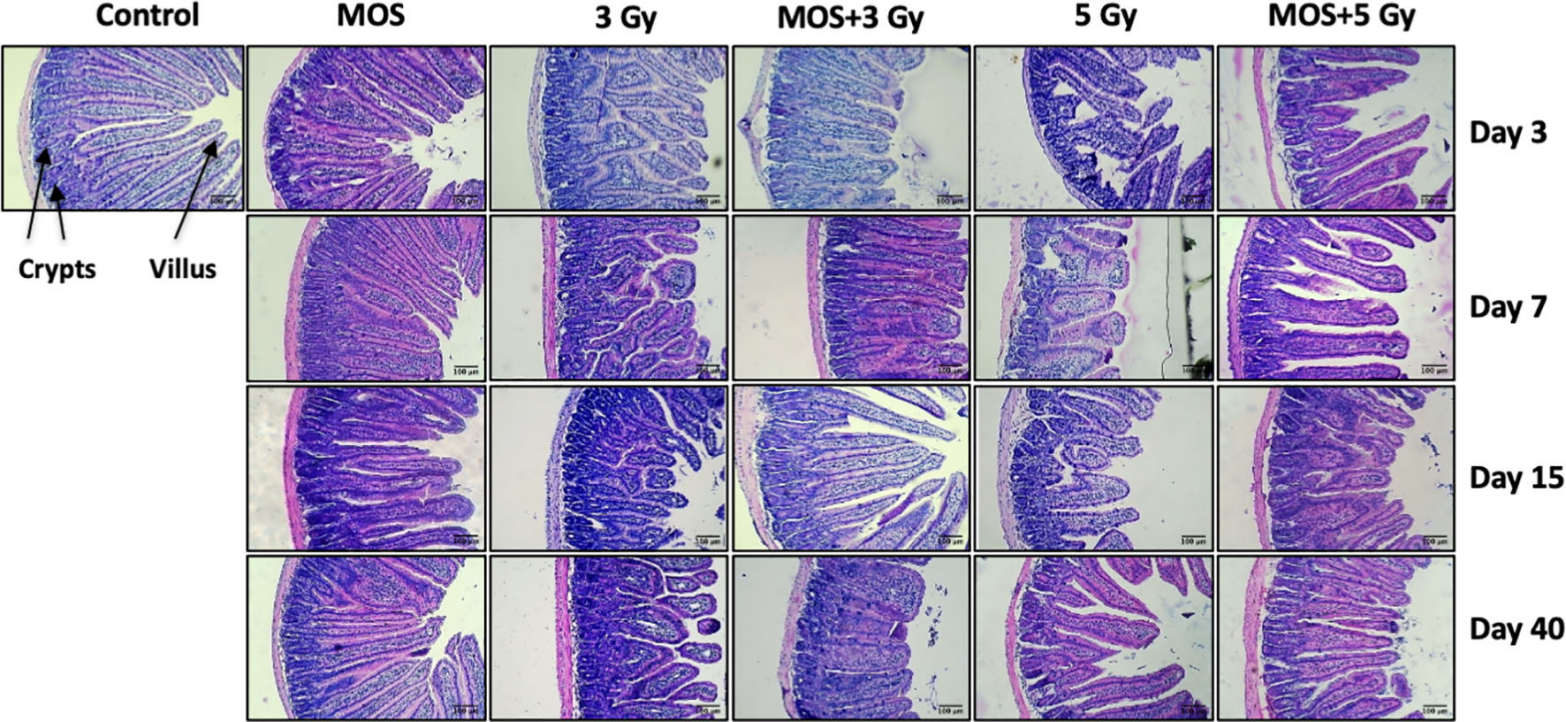
Histological examination of small intestine. Mice were treated with MOS and/or subjected to TBI (3 and 5 Gy) and assessed for histological examination of small intestine on days 3, 7, 15, and 40-post MOS treatment and/or TBI as described in *Materials and Methods*. Scale bar equals 100 μm with an original magnification of ×400.

**Figure 5:**
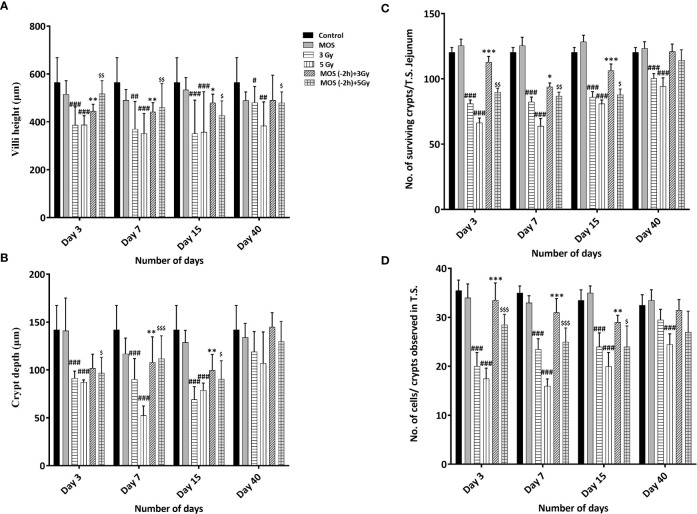
**(A–D)** Histological examination of small intestine: Mice were treated with MOS (200 mg/kg bw) and/or subjected TBI (3 Gy and 5 Gy) and thereafter assessed for histological examinations of small intestine on days 3, 7, 15 and 40-post MOS treatment and/or TBI as described in materials and methods. Representation of **(A)** Villi height (mm) **(B)** Crypt depth (mm) **(C)** No. of surviving crypts/T.S. Jejunum **(D)** No. of cells present per crypt observed in T.S. Error bars represent mean ± SD, and *p < 0.05, **p < 0.01, ***p < 0.001,^#^ *p* < 0.05, ^##^ *p* < 0.01, and ^###^ *p* < 0.001 , ^$^ *p* < 0.05, ^$$^ *p* < 0.01 and ^$$$^ *p* < 0.001 were considered significant, ^#^compared to unirradiated control, *compared to 3 Gy irradiated cohorts and ^$^compared to 5 Gy irradiated cohorts.

H&E stained sections of the jejunum were analyzed on days 3, 7, 15, and 40-post irradiation. Jejuna from MOS treated animals prior to TBI (both 3 and 5 Gy) and irradiated animals (both 3 and 5 Gy TBI) showed shortening of the villi and loss of structural integrity at days 3 and 7 (**Figure 4**). However, the villi blunting and loss of structure were significantly less in MOS-pretreated animals in comparison to the respective irradiated animals. Similarly, there was significant decline in crypt depth post TBI in comparison to un-irradiated control, and the decrease was less in MOS-pretreated animals. MOS pretreated animals showed signs of accelerated recovery as demonstrated by the increase in villi length (427 ± 48.5 μm, *p* < 0.05 and 359 ± 31.09 μm, *p* < 0.05; **Figure 5A**), crypt depth (**Figure 5B**), and the substantial decline in blunting in comparison to the irradiated group (villi length; 237 ± 84.2 μm, *p* < 0.05 and 206.66 ± 23.094 μm, *p* < 0.05, crypt depth) day 15, post 3 and 5 Gy TBI respectively. By day 40 the morphology of jejuna in both irradiated and MOS-pretreated animals had returned to normal. The number of surviving and regenerating crypts per intestinal circumference was scored, and the average per mouse and per group was determined. The average viable crypts per T.S. of jejunum in the untreated control were about 120.5 ± 3.56 (**Figure 5C**). MOS treatment alone exhibited non-significant changes in the number of crypts (125 ± 3.53, 125 ± 6.36, 128 ± 3.6, and 123 ± 4.7) than that of control (on days 3, 7, 15, and 40 respectively). The 3 and 5 Gy cohorts exhibited reduced number of viable crypts to 81 ± 2.82, *p* < 0.001, 82.5 ± 3.53, *p* < 0.001, 86 ± 4.24, *p* < 0.001, 100.5 ± 3.53, *p* < 0.001, and 66 ± 3.53, *p* < 0.001, 64 ± 5.65, *p* < 0.001, 81 ± 2.9, *p* < 0.001, 94.5 ± 6.36, *p* < 0.001 on days 3, 7, 15, and 40 post TBI respectively. Pre-irradiation administration of MOS in 3 and 5 Gy TBI cohorts showed significantly higher number of viable crypts on day 3 (113 ± 4.23, *p* < 0.01 and 90 ± 2.82, *p* < 0.01) and day 7 (94 ± 2.82, *p* < 0.01 and 87± 2.9, *p* < 0.01) respectively in comparison to irradiated control group.

Numbers of cells per crypt were counted and shown in **Figure 5D**. The average cells per crypt of jejunum in the untreated control were about 35.5 ± 2.32. MOS treatment alone exhibited non-significant changes in the number of crypts than that of control in all the time points studied. The number of cells was reduced to 20 ± 2.82, *p* < 0.001, 23.5 ± 2.12, *p* < 0.001, 24 ± 2.9, *p* < 0.001, 29.5 ± 2.12, *ns* and 17.5 ± 2.12, *p* < 0.001, 16 ± 1.41, *p* < 0.001, 20 ± 2.9, *p* < 0.001 24 ± 2.2, *ns* on days 3,7, 15, 40 post (3 and 5 Gy) TBI respectively. MOS pretreatment showed higher number of cells per crypt in MOS + 3 Gy and MOS + 5Gy cohorts on day 3 (113 ± 4.23 and 90 ± 2.82, *p* < 0.05) and day 7 (94 ± 2.82, *p* < 0.01 and 87± 2.9, *p* < 0.01) in comparison to irradiated cohorts respectively. Significant recovery was evident in MOS-pretreated cohorts in terms of villi height, crypt depth, number of surviving crypts, and cells per crypt 15 days post TBI.

### Xylose Absorption Assay

**Figure 6:**
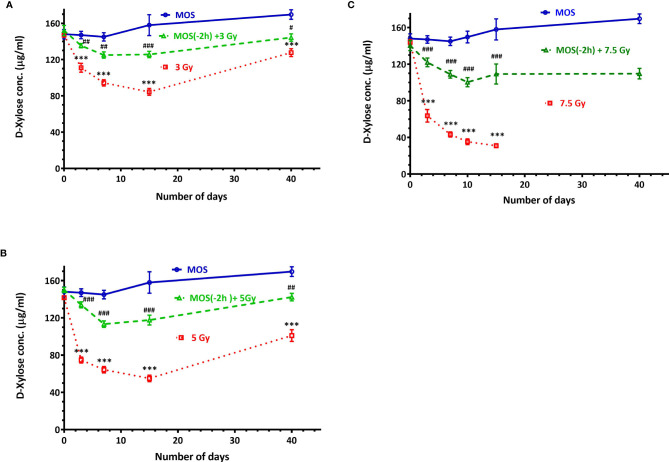
D-Xylose absorption assay: Mice were treated with MOS (200 mg/kg bw) and/or subjected to **(A)** TBI (3 Gy) and assessed for absorptive capacity of the small intestine on days 0, 3, 7, 15, and 40; **(B)** TBI (5 Gy) assessed for absorptive capacity of the small intestine on days 0, 3, 7, 15, and 40; **(C)** TBI (7.5 Gy) assessed for absorptive capacity of the small intestine on days 0, 3, 7, 10, 15, and 40 post TBI as described in *Materials and Methods*. Error bars represent mean ± SD, ***p < 0.001 were considered significant compared to un-irradiated control group and ^###^ *p* < 0.001were considered significant compared to irradiated group.

Functional regeneration and absorptive capacity of the intestines in TBI mice were determined by measuring intestinal absorption of D-xylose. Serum D-xylose level in non-irradiated group was 148 ± 8.88 μg/ml, and there was progressive reduction in xylose absorption in 3 Gy TBI mice (111 ± 8.5 μg/ml, p < 0.01), 5 Gy TBI mice (74.66 ± 5.5 μg/ml, p < 0.001), and 7.5 Gy irradiated mice (63.66 ± 11.84 μg/ml, p < 0.001). MOS pretreatment exhibits significant recovery in the xylose absorption 3 days post TBI in mice exposed to 3 Gy (135 ± 4.16 μg/ml, p < 0.01), 5 Gy (134 ± 5.56 μg/ml, p < 0.001), and 7.5 Gy (112 ± 9.84 μg/ml, p < 0.001); (**Figures 6A–C**). Xylose absorption further declined on day 7 post TBI in the irradiated cohorts 3 Gy (94.33 ± 6.02 μg/ml, p < 0.001), 5 Gy (64 ± 6.02 μg/ml, p < 0.001), and 7.5 Gy (43.44 ± 4.9 μg/ml, p < 0.001) respectively as well as the MOS-pretreated cohorts 3 Gy (125.21 ± 6.32 μg/ml, p < 0.01), 5 Gy (113.41 ± 6.02 μg/ml, p < 0.001), and 7.5 Gy (90.33 ± 9.29 μg/ml, p < 0.001). A time course study (1–40 days) showed significant recovery of xylose absorption in MOS-pretreated cohorts at day 15 post 3 Gy (125.31 ± 6.11 μg/ml, p < 0.001), 5 Gy (117.51 ± 6.02 μg/ml, p < 0.001) 7.5 Gy TBI (102 ± 8.0 μg/ml, p < 0.001) and continued to improve till day 40 post 3 Gy (142.31 ± 7.03 μg/ml, p < 0.001), 5 Gy (144.51 ± 7.02 μg/ml, p < 0.001), and 7.5 Gy TBI (109.37 ± 19 μg/ml, p < 0.001) thereby, indicating the functional regeneration of intestine after radiation injury. The 7.5 TBI cohorts exhibited surplus decline in xylose absorption at day 10 (35.33 ± 5.51 μg/ml, p < 0.001) and day 15 (31 ± 3.6 μg/ml, p < 0.001) with no surviving animals in the group after day 15. The 7.5 Gy TBI animals were incapable of demonstrating adequate xylose absorption after radiation injury, further contributing to animal mortality. The 3 Gy (84 ± 6.65 μg/ml, p < 0.001) and 5 Gy (55.51 ± 6.32 μg/ml, p < 0.001) TBI cohorts exhibited further decline in the intestinal absorptive capacity on day 15, with improvement in xylose absorption on day 40 (127.67 ± 7.50 μg/ml, p < 0.001) and (101.82 ± 11.21 μg/ml, p < 0.001) respectively.

### MOS Pretreatment Lowers MDA Levels in the Kidney and Liver Mitochondria

Ionizing radiation causes extensive damage to lipid component of biological membrane due to initiation of chain reaction of free radical formation (31). Phospholipids along with protein components of the biological membrane are also equally vulnerable to the free radical mediated attack resulting in oxidative modification rendering them non-functional (25, 32). Lipid peroxidation leads to formation of mutagenic MDA, compromising membrane fluidity, integrity, and biological functioning.

**Figure 7:**
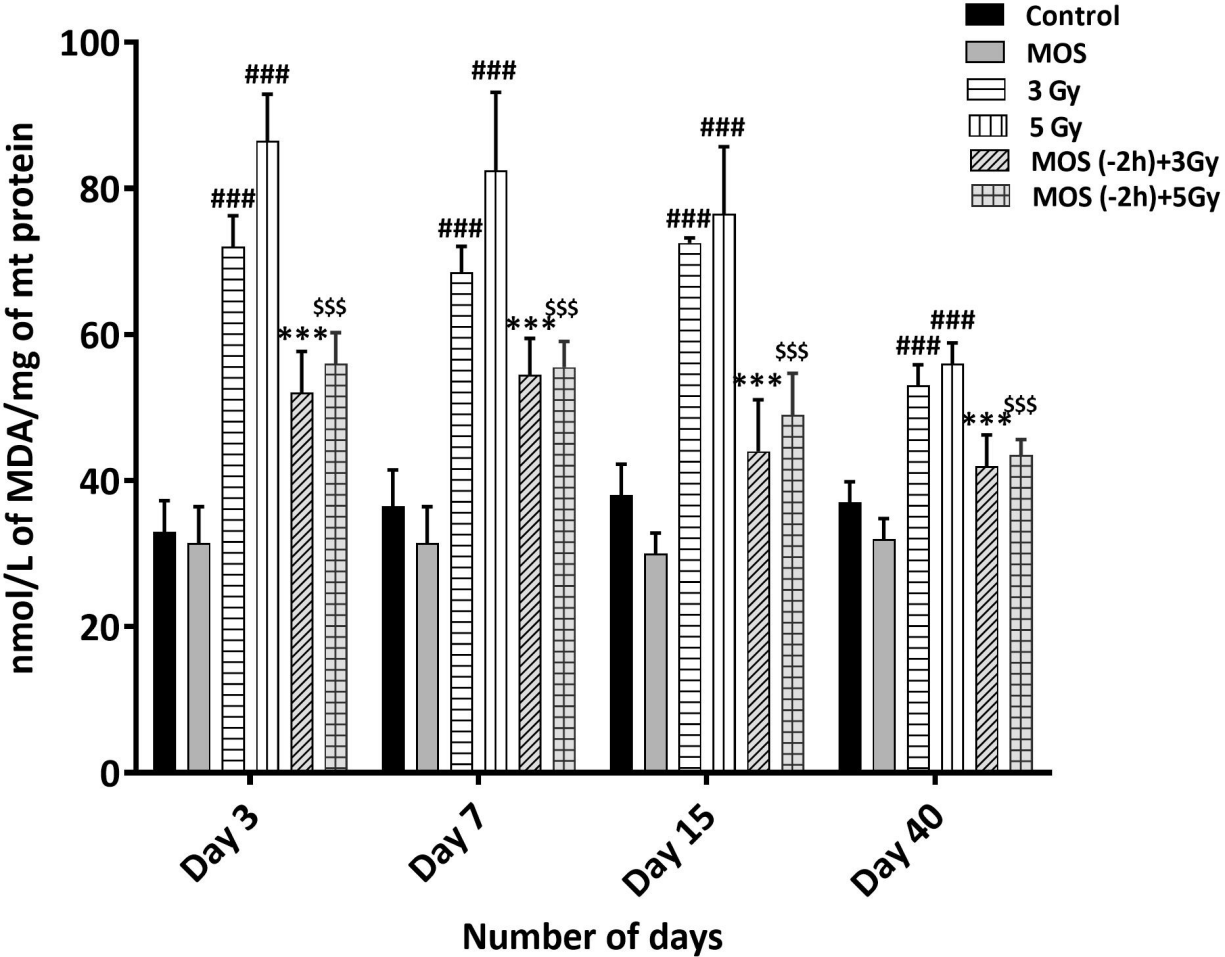
Lipid peroxidation assay: Mice were treated with MOS (200 mg/kg bw) and/or subjected to TBI (3 and 5 Gy) and assessed for MDA levels in mice kidney mitochondria as described in *Materials and Methods*. Error bars represent mean ± SD, ^###^ *p* < 0.001 were considered significant compared to un-irradiated control group, ***p < 0.001 were considered significant compared to irradiated group and ^$$$^ *p* < 0.001 were considered significant compared to 5 Gy irradiated group.

Results show intensive increase in MDA concentration 3 days post 3 and 5 Gy TBI mice kidney (72 ± 4.24, *p* < 0.001 and 86.5 ± 6.36, *p* < 0.001 nM/L/mg of mitochondrial protein) in comparison to un-irradiated control (33 ± 4.2, *p* < 0.001); **Figure 7**. MDA concentration remained significantly high in TBI mitochondria (of mice kidney). MOS pretreatment in combination cohorts has shown significant decrease in MDA levels in the kidney mitochondria in comparison to that of irradiated animals at all the studied time points.

### MOS Administration Prior to TBI Ameliorates Free Thiol Levels in Mitochondria

**Figure 8:**
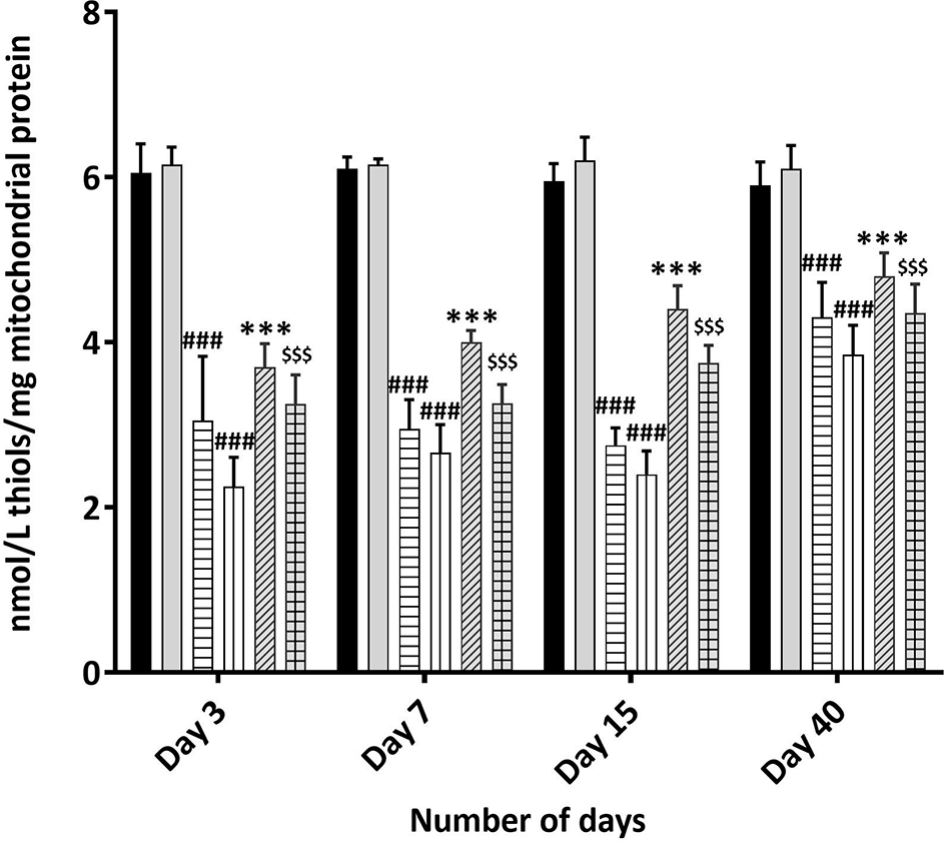
Total Thiol estimation using Ellman’s reagent: Mice were treated with MOS (200 mg/kg bw) and/or subjected TBI (3 and 5 Gy) and assessed for total thiol levels in mice kidney mitochondria as described in *Materials and Methods*. Error bars represent mean ± SD, ^###^ *p* < 0.001 were considered significant compared to un-irradiated control group, ***p < 0.001 were considered significant compared to irradiated group and ^$$$^ *p* < 0.001 were considered significant compared to 5 Gy irradiated group.

Presence of thiols in the biological system is of great significance in redox metabolism both as components of protein structures and as metabolic intermediates. Protein thiols play an important role in many cellular functions, including protein folding, enzyme catalysis, and metabolic regulation. Thiols are present as protein thiols and non-protein thiols and have ability to directly scavenge free radicals. Exposure to IR is known to deplete GSH and other thiols in animal tissues in dose dependent manner. Therefore, measurement of the biological thiols is important to investigate their roles in IR induced oxidative stress. Results show dramatic decrease in thiol concentration 3 days post 3 and 5 Gy TBI mice kidney (3.05 ± 0.778, *p* < 0.001 and 2.25 ± 0.354, *p* < 0.001 nM/L/mg of mitochondrial protein) in comparison to un-irradiated control (6.050 ± 0.35, *p* < 0.001); **Figure 8**. Acid soluble thiol levels remained significantly lesser in TBI mitochondria (of mice kidney) at all time points studied in comparison to that of control. MOS pretreatment in combination cohorts has shown significant increased thiol levels in the kidney mitochondria in comparison to that of irradiated animals at all the studied time points. MOS administration prior to TBI improved thiol levels in mitochondria (kidney) of animals in comparison to that of irradiated group.

## Discussion

**Figure 9:**
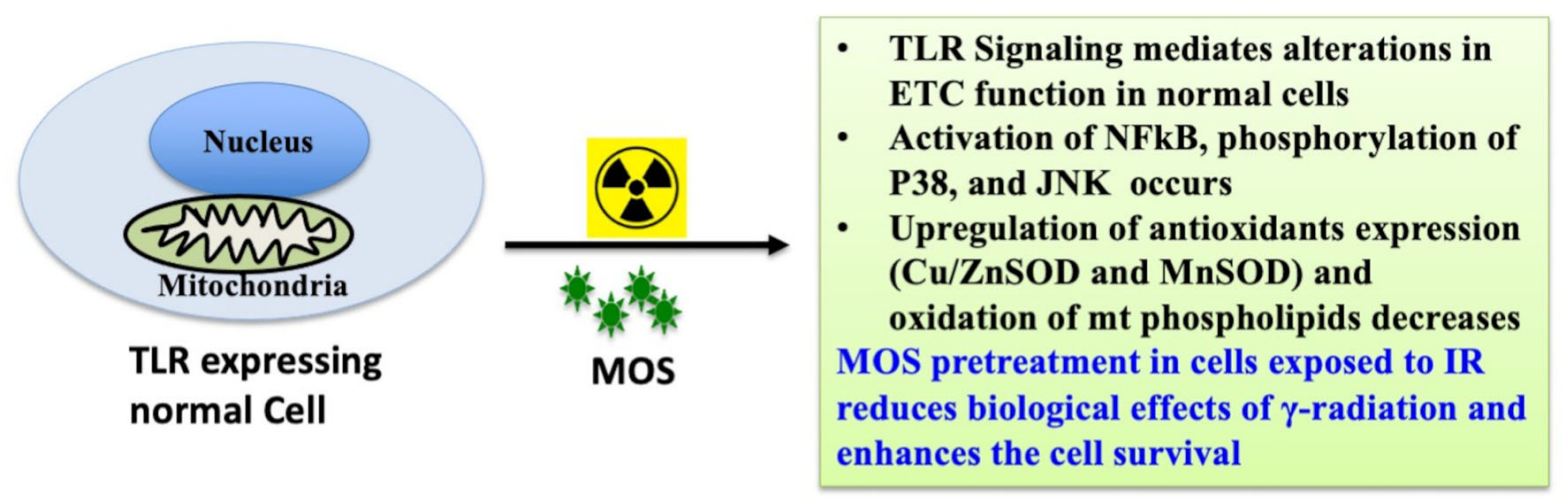
MOS mediated radiation protection *in vitro*: MOS mediated radiation protection in normal cells requires TLR and ETC function. Under *in vitro* system, mannan counters radiation response through activation of NF*κ*B, p38 and JNK, alteration in mitochondrial physiology, increase in MnSOD and Cu/ZnSOD expression, and decrease in peroxidation of cardiolipin inside mitochondrial inner membrane.

Prebiotic MOS is reported to have beneficial effects in overall health of an organism, possess intestinal stimulatory capability, and immune modulation potential. And we have earlier reported *in vitro* radiation protection efficacy of MOS in normal cells with intact respiratory function (**Figure 9**) (19). The present study focuses on demonstrating radioprotective effects of MOS, a prebiotic and understanding its role in reducing deleterious effects of TBI on hematopoietic and gastrointestinal system. Mannan pretreatment (IP 50 mg/kg BW or oral 200 mg/kg BW, separately) confers 100% survival advantage to 8–2 weeks old mice (both male and female) against lethal dose of TBI (7.5 Gy) compared to 100% mortality within 14 days after IR exposure alone group (**Figures 1A, B**). Moreover, improved survival (approximately 62 and 50%) in MOS pretreated BALB/c mice (12–13 months old female and male mice respectively) receiving lethal dose (7.5 Gy) of TBI was also observed (**Figure 1D**). There was less decline and consequently prompt recovery of the radiation-induced loss of body weight in MOS pretreated cohort post total body irradiation (TBI) in comparison to irradiated animals (data not shown).

Hematopoietic stem cells and intestinal crypt stem cells are extremely sensitive to damage by IR because of their highly proliferative nature (30). Sensitivity of bone marrow against TBI has been well documented, and pancytopenia is a major factor in radiation-induced morbidity and mortality (33). We assessed the effects of MOS pretreatment on bone marrow and radiation-induced suppression in the number of circulating blood cells by exposing mice to 3 and 5 Gy TBI at days 3, 7, 15, and 40. There was massive ablation of cellular content of the bone marrow and consequent decrease in number of circulating blood cells after 3 and 5 Gy TBI (**Figures 2A–E**). Damage to hematopoietic stem cells results in depletion of peripheral blood lymphocytes and consequently enhances the susceptibility of organism to opportunistic secondary infections. Results reveal reduction in various hematological parameters post TBI, and the baseline levels for most were never achieved in TBI mice. On the other hand, MOS modulated rapid recovery of the constituent parameters leading to attainment almost baseline levels of most peripheral blood cells by day 30 (**Figures 2A–E**). MOS pretreatment in mice reduced radiation induced damage to hematopoietic stem cells and instigated rapid restoration of bone marrow cellular content (**Figure 3**) resulting in substantial increase in the number of circulating blood cells (**Figures 2A–E**). Stimulation of hematopoietic stem cells might be involved in promoting prompt recovery of hematopoietic system in comparison to irradiated group. IR poses damage to both gastrointestinal system and hematopoietic system and both of these losses can independently or synergistically result in mortality. It has been shown that doses that manifest GI-ARS also impact bone marrow tremendously and lower ability of the body to manage the infections caused by intrusions in intestinal mucosal barrier (34). Contrarily, findings from another study clearly separate the effects of radiation on the GI epithelium from those on the BM or BM-derived cells and have shown that the radio-sensitivity of the BM does not influence radio-sensitivity of the GI epithelium (30). The effects of MOS pretreatment on the radiation-induced damage to the GI system were analyzed by histological examination of the jejunum on days 3, 7, 15, and 40 post TBI. Results depict that MOS pretreatment followed by TBI minimizes crypt cell death and denudation of villi in comparison to the respective irradiated animals. Moreover, MOS pretreated animals showed signs of accelerated overall recovery as demonstrated by substantial decline in blunting, early restoration of normal morphology, and absorption function of small intestine (**Figures 4** **–** **6**).

**Figure 10:**
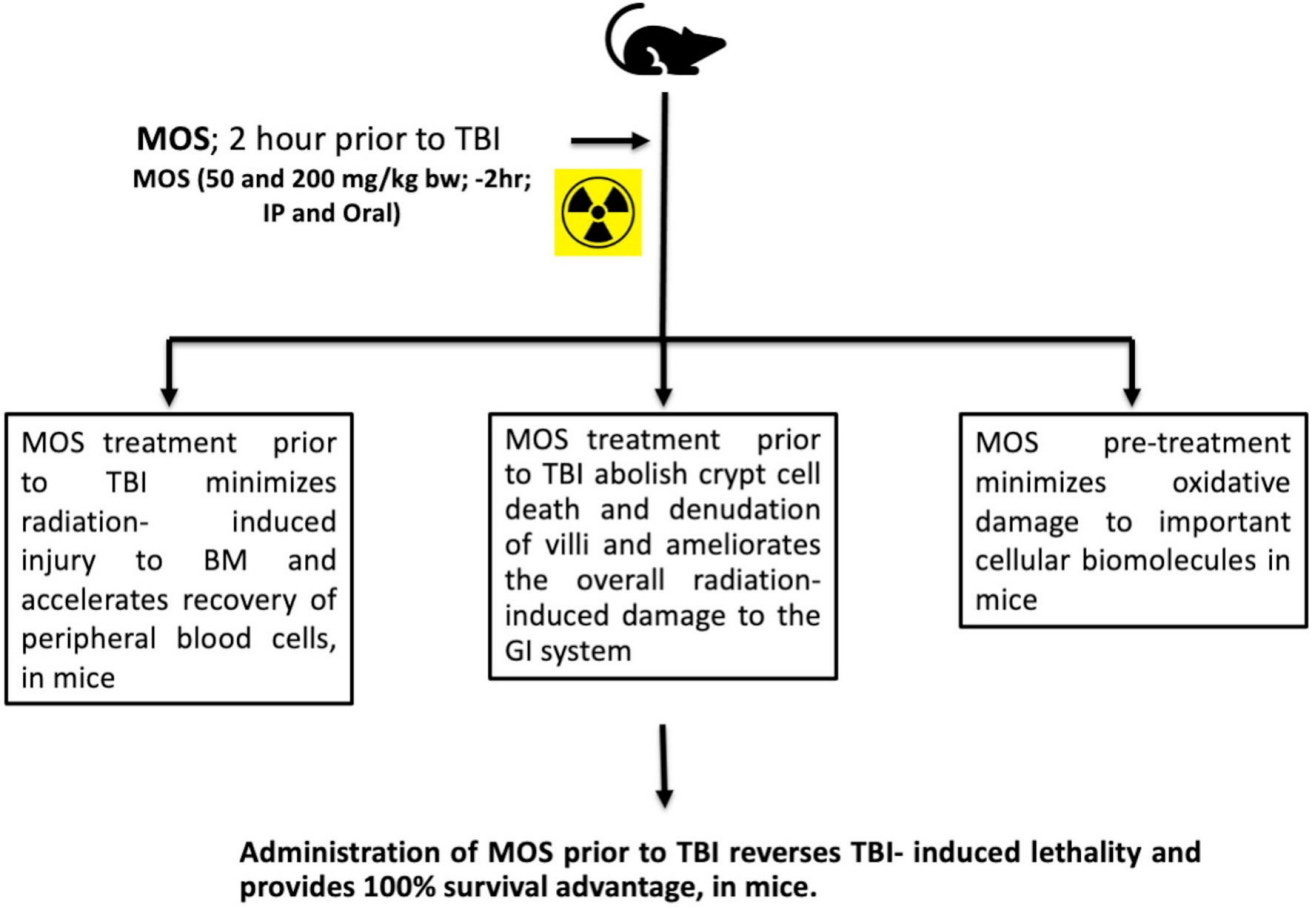
MOS mediated radiation protection *in vivo*: Under *in vivo* condition, MOS pretreatment abates radiation-induced damage to hematopoietic system and gastrointestinal system, accelerates recovery, and consequently abrogates TBI-induced lethality in mice.

Presence of thiols in biological system is of great significance in redox metabolism both as components of protein structures and as metabolic intermediates (35). Thiols play an important role in many cellular functions, including protein folding, enzyme catalysis, and metabolic regulation (36–38). Thiols have the ability to directly scavenge free radicals and may act as cofactor for enzymes involved in management of oxidative stress (38, 39). Exposure to IR is known to deplete GSH and other thiols in animal tissues in a dose dependent manner. Therefore, we measured acid soluble thiols to investigate their roles in IR induced oxidative stress. MOS administration prior to TBI ameliorated acid soluble thiol levels in mitochondria (of liver and kidney) of animals in comparison to that of irradiated group (**Figure 8**). Ionizing radiation causes extensive damage to lipid component of biological membrane due to initiation of chain reaction of free radical formation (31). Phospholipids along with protein components of the biological membrane are also equally vulnerable to the free radical attack resulting in oxidative modification rendering them non-functional (40). Lipid peroxidation leads to formation of mutagenic MDA, compromising membrane fluidity, integrity, and biological functioning (41). MOS pretreatment in combination cohorts has shown significant decrease in MDA levels in the kidney and liver mitochondria in comparison to that of irradiated animals (**Figure 7**). Mitochondria is an important cell organelle that is both a potential source as well as target of ROS. Correspondingly, under *in vitro* system, we have demonstrated that MOS counters IR induced ROS through alteration in mitochondrial physiology, increase in MnSOD and Cu/ZnSOD expression, and decrease in peroxidation of cardiolipin inside mitochondrial inner membrane (19). As aging progresses, there is more damage accumulation in mitochondria, and there is decline in electron transport chain function. Since mitochondrial respiratory capacity is known to decline with age, reduction in survival percent in mannan pretreated aged mice (50–60% in 12–13 months old mice with respect to 100% survival in 8–12 weeks old mice) was well expected after TBI. Another independent study was done in our laboratory to assess the effects of ionizing radiation on GI tract microflora in MOS treated mice post TBI. Mice gut microflora was cultured from the fecal matter followed by characterization of bacteria based on morphology and differential staining. The gut microflora largely remains unperturbed in MOS pretreated mice, and there was decline in microflora type and number in TBI mice. The results of the study will be discussed in detail elsewhere (unpublished data). Results demonstrate that MOS ameliorates the radiation-induced damage to the hematopoietic and GI system, accelerates recovery leading to enhanced animal survival demonstrating its protection efficacy against TBI induced mortality (**Figure 10**).

In summary, MOS treatment prior to TBI effectively minimizes radiation-induced hematopoietic and gastrointestinal injury, accelerates recovery of circulating blood cells, minimizes oxidative damage to important cellular biomolecules, restores intestinal integrity, and consequently abrogates TBI-induced lethality. Earlier, we have shown that TLR and mitochondrial ETC functions are inevitable in radio-protective efficacy exhibited by mannan. These observations clearly demonstrate the potential of MOS as a countermeasure agent to ameliorate biological effects of radiation. Abrogation of damage to both GI and hematopoietic system may play a major role in enhanced recovery of organism and thereby improved overall survival. Further studies are necessary to unravel the mechanisms underlying decrease in the radiation-induced damage to stem cell compartments of tissue and accelerated repair of tissue damage by mannan pretreatment, besides its ability to abolish cellular oxidative stress. In recent years, numerous radiation countermeasure agents have been reported, most of which are efficient but relatively toxic. MOS, as a radiation countermeasure agent, may be beneficial in case of planned radiotherapy events. However, the present study was done on single strain of mice model, and the results need to be validated in other more suitable animal model at pre-clinical levels.

## Data Availability Statement

The original contributions presented in the study are included in the article/supplementary material. Further inquiries can be directed to the corresponding author.

## Ethics Statement

The animal study was reviewed and approved by the Animal Ethical Committee, Institute of Nuclear Medicine & Allied Sciences (INMAS), Defence Research and Development Organization (DRDO).

## Author Contributions

SS and DG designed research, performed research, analyzed data, and wrote the paper. All authors contributed to the article and approved the submitted version.

## Funding

This study was supported by Defence Research and Development Organization (DRDO), Ministry of Defence, India.

## Conflict of Interest

The authors declare that the research was conducted in the absence of any commercial or financial relationships that could be construed as a potential conflict of interest.

## Abbreviations

IR, ionising radiation; ARS, acute radiation syndrome; MOS, mannan oligosaccharide; H-ARS, hematopoietic acute radiation syndrome; GI-ARS, gastrointestinal acute radiation syndrome; HSCs, hematopoietic stem cells; TBI, total body irradiation; TLR, toll-like receptor; RCAs, radiation countermeasure agents; BM, bone marrow.
